# Use of artificial intelligence in discerning the need for prostate biopsy and readiness for clinical practice: a systematic review protocol

**DOI:** 10.1186/s13643-023-02282-6

**Published:** 2023-07-17

**Authors:** Elisa Martinez-Marroquin, Minh Chau, Murray Turner, Hodo Haxhimolla, Catherine Paterson

**Affiliations:** 1 Faculty of Science and Technology, University of Canberra, Canberra, Australian Capital Territory 2617 Australia; 2 Prehabilitation, Activity, Cancer, Exercise and Survivorship (PACES) Research Group, Faculty of Health, University of Canberra, Canberra, ACT 2617 Australia; 3 Robert Gordon University, Aberdeen, AB10 7AQ Scotland, UK

**Keywords:** Artificial intelligence, Prostate cancer, Diagnosis, Diagnostic pathway, Systematic review protocol, Technology maturity level, AI adoption readiness

## Abstract

**Background:**

Variability and inaccuracies in the diagnosis of prostate cancer, and the risk of complications from invasive tests, have been extensively reported in the research literature. To address this, the use of artificial intelligence (AI) has been attracting increased interest in recent years to improve the diagnostic accuracy and objectivity. Although AI literature has reported promising results, further research is needed on the identification of evidence gaps that limit the potential adoption in prostate cancer screening practice.

**Methods:**

A systematic electronic search strategy will be used to identify peer-reviewed articles published from inception to the date of searches and indexed in CINAHL, IEEE Xplore, MEDLINE, Scopus, and Web of Science Core Collection databases. Registries including Cochrane Central Register of Controlled Trials, ClinicalTrials.gov and International Clinical Trials Registry Platform (ICTRP) will be searched for unpublished studies, and experts were invited to provide suitable references. The research and reporting will be based on Cochrane recommendations and PRISMA guidelines, respectively. The screening and quality assessment of the articles will be conducted by two of the authors independently, and conflicts will be resolved by a third author.

**Discussion:**

This systematic review will summarise the use of AI techniques to predict the need for prostate biopsy based on clinical and demographic indicators, including its diagnostic accuracy and readiness for adoption in clinical practice.

**Systematic review registration:**

PROSPERO CRD42022336540

## Background

Prostate cancer is the most prevalent form of cancer among men [1–3], and the primary screen for prostate cancer is the prostate-specific antigen (PSA) blood test and digital rectal examination (DRE). When cancer is suspected, a needle biopsy under general or local anaesthetic is performed to confirm the diagnosis. Prostate biopsies are expensive and require an invasive and undignified procedure which can cause complications, such as pain, bleeding, infection, and post procedure anxiety. In addition, some men experience stress because of lengthy wait-list times in accessing a prostate biopsy service and further wait times in receiving the biopsy results [4–6]. Notably, while prostate cancer is a common diagnosis, the mortality rate from this disease is low [7]. Therefore, patients with non-life-threatening cancer^1^ are closely monitored, sparing them from the bothersome and distressing side effects of therapy which negatively impacts health-related quality of life [8, 9]. These clinical considerations underscore the importance of limiting the biopsies and treatment to men with aggressive cancer. However, currently, a significant number of men undergo prostate biopsies to find indolent cancer or no cancer at all. This has triggered research to better utilise pre-biopsy tests and the adoption of several prostate cancer risk calculators to reduce the number of biopsies and treatment when they are not strictly necessary.

Although a high PSA value is associated with an increased risk of prostate cancer, the PSA threshold to recommend a biopsy remains controversial. The main reason is that benign pathologies such as benign prostate enlargement, urine infections, or prostatitis can also cause high PSA levels. Besides, clinical studies have shown that prostate cancer can be detected even with low PSA levels. A transrectal ultrasound-guided prostatic biopsy (TRUS) is the standard to confirm the diagnosis in patients with elevated PSA [10]. However, it has a significant false-negative rate (risk of missing an aggressive cancer) and false-positive rate (risk of overdiagnosing clinically insignificant cancer) [11, 12]. Notably, overtreatment carries the very real risk of compromising the patient’s quality of life, particularly in urinary, bowel, and sexual dysfunction [13]. This highlights the importance of ensuring men receive an accurate diagnosis of potentially life-threatening cancer while simultaneously avoiding unnecessary invasive biopsies and therapy in men with clinically insignificant cancer. The multiparametric magnetic resonance imaging (mpMRI) was introduced to increase the diagnostic accuracy and reduce the number of biopsies, but the interpretation of mpMRIs has proven to be dependent on the reader’s training and experience [14, 15]. Due to the problems with variability between readers and reporting, this has slowed progress and prevents a consistent improvement in the efficiency and accuracy in the diagnostic pathway [16–18].

Several other non-invasive indicators have been proposed in recent years, and evidence is mounting that these pre-biopsy indicators may embed enough information for the prediction of life-threatening cancer [19–24]. Research is emerging aiming at detecting clinically significant prostate cancer based on the interpretation of a range of these diagnostic indicators, such as PSA, 4Kscore, prostate size, age, clinical history, prostate-specific membrane antigen (PSMA), PET scan (positron emission tomography), and multiparametric MRI (mpMRI) scan (T1, T2 score, DWI score, DCE score, and PIRADS score). The final assessment is dependent on the ability of the health professional to combine and interpret the available information. Overall, inaccuracies in the diagnosis are extensively documented in the research literature [12], and a more standardised and accurate approach to assess the results of the noninvasive diagnostic tests is urgently needed to advance the field [16, 25, 26]. Further research is required to identify a more accurate selection of patients for biopsy and to assist clinicians to counsel patients more accurately in the complex decision-making process in the continuum of prostate cancer care [8, 27].

The combined assessment of multiple parameters is challenging for the human specialist and may present large variability between specialists, depending on their training and level of experience. As a result, it is still challenging to assess the clinical significance of prostate cancer accurately prior to the biopsy [28]. In order to increase the diagnosis objectivity and accuracy, the use of statistical analysis and computer-aided tools based on AI has been explored [29, 30]. AI uses complex models able to capture the high-dimensional relationships among the input parameters that influence the outcome, beyond the ability of human experts. There has been a previous systematic review [31] which aimed to explore the role of AI to improve broadly the diagnosis and management of prostate cancer. There are several limitations to point out in this systematic review: (1) there was no methodological quality assessment of the included studies conducted; therefore, the current state of the evidence is problematic to discern; (2) the inclusion and exclusion criteria are not clearly defined; although other AI methods are listed as keywords, all the included manuscripts use only artificial neural networks; and (3) the review is clinically outdated by year of publication given the literature search dates were not reported either.

A contemporary scoping review has summarised recent machine learning (ML) and deep learning (DL) applications specifically in prostate MRI lesion analysis [32]. The use of AI in the overall diagnostic decision may have promising results by using a combination of diagnostic indicators to predict aggressive prostate cancer. Capturing existing insights which incorporates the patients and clinicians’ perspectives is also important in the examination of the clinical acceptability of the AI-assistive systems in prostate cancer detection which has not been considered in a previous systematic review [31, 32]. To advance the smart integration of cutting-edge research in AI, consideration to connect expertise in prostate cancer multidisciplinary teams is essential for adoption of AI in clinical practice in the future.

**Fig. 1:**
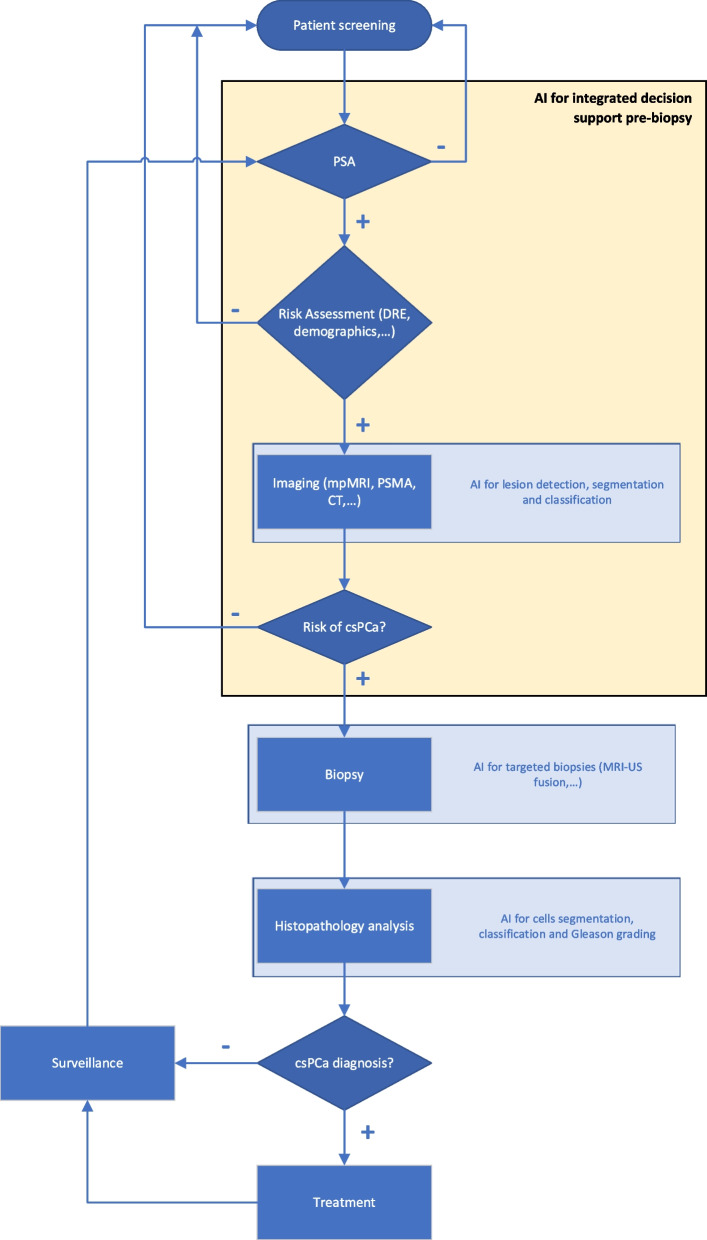
Prostate cancer diagnostic pathway process map. Process map for prostate cancer diagnosis in biopsy-naïve patients with use of AI at different stages of the diagnostic pathway

The aim of this systematic review is to analyse how AI has been used in the prostate cancer diagnostic pathway for integrated decision support in biopsy-naïve patients (see Fig. 1), to summarise the results that have been obtained so far, and to assess the readiness for adoption in clinical practice.

This systematic review will address the following research question:

- How has AI been used in predicting patient selection for prostate biopsy based on clinical and demographic indicators and its impact on clinical practice?

## Relevance

A preliminary search of PROSPERO, MEDLINE, and the Cochrane database of systematic reviews was performed, and this yielded no systematic reviews which have previously explored this research question. To date, a significant number of men have undergone biopsy of the prostate as part of a standardised clinical diagnostic pathway to find a negative or indolent cancer which does not require treatment and is unlikely to pose any threat to quantity and quality of life [15, 33]. Yet, prostate biopsies are invasive, expensive, and carry serious health risks, such as bleeding, pain, and life-threatening infections [5, 34, 35]. This underscores the strong clinical interest in predicting clinically significant prostate cancer using a wide range of clinical and demographic information so that invasive biopsies are avoided when they are not strictly necessary [23].

A range of studies have emerged in recent years to improve patient selection for prostate biopsy based on pre-biopsy clinical indicators, which are yet to be pooled and critically synthesised. Each use a different set of indicators, different methods to combine them, different patient cohorts to test them, and different assessment of results. The findings are promising but not yet conclusive. There is a need to summarise the different initiatives and progress so far and to provide a comparative framework to inform further work. Although AI literature has reported promising results more broadly in cancer, research is needed on the identification of evidence gaps that limit or may facilitate the potential adoption of AI-informed prostate cancer diagnostic pathways, specific to biopsy practices.

## Methods

The protocol for this review was submitted by the authors for registration in PROSPERO (CRD42022336540) prior to the beginning of the review. The review will be carried out according to the Cochrane Handbook for Systematic Reviews of Interventions and reported in accordance with Preferred Reporting Items for Systematic Reviews and Meta-Analysis (PRISMA) guidelines [36].

### Information sources and search strategy

This systematic literature review aims at identifying published and unpublished studies reporting the use of AI for patient selection for prostate biopsy, based on a selection of clinical and demographic indicators.

For this purpose, we will search the following electronic databases from inception to the date searches are run: CINAHL, IEEE Xplore, MEDLINE, Scopus, and Web of Science Core Collection. To increase the inclusiveness of the searches, the following registries, Cochrane Central Register of Controlled Trials, ClinicalTrials.gov, and International Clinical Trials Registry Platform (ICTRP), will be searched to locate unpublished studies. Experts will be invited to provide suitable references.

The search strategy will include a range of keywords to retrieve the concepts of AI, prostate cancer, and diagnostic tests. Search terms will be translated for use in each database, and where available, subject headings (MeSH headings) will also be utilised to increase the sensitivity of searches (Table 1). The searches will be re-run just before the final analyses and further studies retrieved for inclusion. Relevant systematic reviews and reference lists of the included studies will be scrutinised for potentially relevant studies.

**Table 1:** Search strategy

Search no	Concept	Search terms
#1	Artificial intelligence	"Artificial Intelligence + " [MeSH] OR "Computer Heuristics" [MeSH] OR Algorithm* OR “artificial intelligen*” OR AI OR “computational intelligen*” OR “computer-aided” OR “computer assisted” OR “computer heuristic*” OR “computer reasoning” OR “computer vision” OR “deep learning” OR “machine intelligen*” OR “machine learning” OR “neural network*” OR “supervised learning” OR “unsupervised learning”
#2	Prostate cancer	“Prostatic Neoplasms + ” [MeSH] OR “prostatic neoplasm*” OR “prostate neoplasm*” OR “prostate cancer*” OR “prostatic cancer*” OR “cancer of the prostate” OR PCa
#3	Clinical significance	aggressive OR "clinically significant" OR "cancer involvement per core" OR "gleason score* OR ((number OR percentage OR proportion) NEAR/5 (“positive core*” OR “cores positive”))
#4	Diagnosis/screening	"Diagnostic Techniques and Procedures + " [MeSH] OR "Diagnosis + " [MeSH] OR "Diagnosis, Computer-Assisted + " [MeSH] OR "Early Diagnosis + " [MeSH] OR assess* OR classif* OR detect* OR diagnos* OR identif* OR screen*
#5	Use of artificial intelligence in diagnosis of clinically significant prostate cancer	#1 AND #2 AND #3 AND #4

### Study selection

Following de-duplication, the titles and abstracts identified for eligibility will be independently screened by at least two authors of the review team. The full text of all potentially eligible records will be then retrieved and screened independently by two review authors using a data extraction form, linking together multiple records of the same study in the process. Any disagreement will be resolved by discussion or consulting a third review author. The selection process will follow the PRISMA statement guidelines [36]. The screening process will be performed using Covidence systematic review software (Veritas Health Innovation, Melbourne, Australia. Available at http://www.covidence.org). Only studies finally selected will progress to the data extraction stage. There are no limits to language or year of publication.

### Eligibility

#### Types of technology

AI is a broad field that has evolved and expanded in recent years. For this review, we define AI as the suite of techniques that allow the automation of activities associated with human thinking such as decision-making, pattern recognition, or learning.

This systematic review will analyse the use of AI techniques without restrictions. However, given that the problem to be solved is the diagnosis of prostate cancer, the focus is expected to be in classification techniques based on supervised or unsupervised machine learning, such as decision trees, rule-based classifiers, logistic regression, artificial neural networks, support vector machines, Bayesian classifiers, random forest, and deep learning.

#### Types of studies

The primary focus of included studies will be on the use of AI in prostate cancer screening without restrictions on the research design. All quantitative, qualitative, and mixed-method studies will be eligible, irrespective of the research design.

The review will summarise evidence on the use of AI in identifying patients’ clinical indication for a prostate biopsy. Studies will be included if they use an AI technique in the diagnostic pathway for prostate cancer detection up to the stage of prostate biopsy only. This review is focused solely on the use of AI for prostate biopsy patient selection based on more than one clinical and demographic indicator. Studies will be included if they evaluate the performance of the AI technique in a systematic way, including its diagnostic accuracy.

We will exclude studies on the use of AI post biopsy and also studies to improve accuracy of individual diagnostic tests, where AI is applied to standalone steps in the diagnostic pathway, for instance, deep learning for tumour detection [19], grading of prostatectomies [37], and grading of prostate cancer on biopsies’ histopathology images [38, 39].

We will also exclude studies on simulations rather than real patient cohorts, case reports, reviews, commentaries, editorials, and conference abstracts and studies with no clear detail on the AI strategy to avoid unnecessary biopsies and to reduce the overdiagnosis of clinically insignificant prostate cancer.

#### Types of participants

All men diagnosed with a suspected prostate cancer irrespective of pre-biopsy clinical or demographic characteristics.

### Outcomes

The systematic review will allow the identification of the AI techniques most used in the prostate cancer diagnostic pathway to discern between clinically and non-clinically significant cancer in biopsy-naïve patients. The review will investigate how AI is used, including the indicators used as inputs to the AI system, such as PSA, 4kscore, prostate size, and mpMRI PIRADs score. The analysis will provide insight into how the performance of the AI techniques is assessed in this context.

As secondary output measure, we will assess the effectiveness of these techniques and their impact on clinical practice. This review will capture clinical outcomes related to numbers of biopsies performed and avoided, detection of clinically significant cancer, and outcomes related to the views among members of the multidisciplinary prostate cancer team on the use of AI in prostate cancer clinical practice.

### Data extraction and management

Two review authors will independently extract outcome data. Study characteristics will be extracted by one review author, and a second review author will check data extractions for accuracy. A data extraction form will be developed and piloted before its use on several studies and agreed in the review team prior to completing all data extraction. In the case of incomplete reported data, study authors will be contacted.

Data to be extracted will capture the characteristics of included studies including the following: authors, year of publication; country of origin; study aim and study design; clinical and demographic variables, ground truth indicator or target output for diagnostic accuracy; countries and institutions where the patients’ data were collected; the numbers of participants who were included in the study; losses and exclusions of participants, with reasons; AI technique used; clinical performance measure(s); view of AI in clinical practice from end users; and evidence gaps for adoption in clinical practice.

### Methodological quality assessment

The final retained full-text studies will undergo methodological quality assessment independently by two reviewers and any disagreements resolved by discussion. None of the studies will be excluded based upon their methodological quality score to enable a comprehensive overview of the current state of the evidence. The methodological quality assessment will be conducted using the mixed-methods assessment tool (MMAT). The MMAT was selected because it enables a plethora of study designs to methodological appraised given the integrative review design. This assessment tool enables critical appraisal of all qualitative, quantitative, and mixed-methods studies. Each domain of assessment is rated against, “no”, “yes”, and “unclear”. Methodological quality assessment will be performed by one reviewer and quality checked by a second reviewer. In the case of diagnostic accuracy studies, the reviewers will use the quality assessment of diagnostic accuracy studies (QUADAS)-2 quality checklist. Briefly, the included studies will be assessed in four main key domains: (1) patient selection, (2) index test, (3) reference standard, and (4) flow and timing.

### Data synthesis and results

A meta-analysis will be performed to calculate pooled diagnostic accuracy measures if data allows. If this is not possible, then a narrative synthesis will be used. The steps in the narrative synthesis will involve the following: (1) data reduction by tabulation, (2) data comparison between studies, and, finally, (3) drawing conclusions. This process involves reading the full papers multiple times, linking together similarities and differences between the studies, and quality checking with the primary sources. The data reduction involves delineation of outcomes. The data comparison phase involves the reviewers’ identifying patterns and themes through counting and clustering and contrasting the study findings. Finally, the drawing of conclusions and verification inform a comprehensive understanding of the topic, which will be verified with the primary sources of data for accuracy throughout the process. The data synthesis will be conducted by two reviewers and consulted with a third reviewer. The narrative synthesis of diverse research designs will be reported according to the Synthesis without Meta-analysis (SWiM) in systematic reviews [40]. The Cochrane Review Manager will be used for primary analyses, including risk of bias, and Meta-DiSc software for the meta-analysis of test accuracy data and for secondary analyses [41]. The index test used to evaluate the solutions proposed in the included papers is based on a binary outcome (presence of or absence of malignant condition). A univariate random-effects model will be employed to obtain summary estimates of the sensitivity and specificity of the test.

**Fig. 2:**
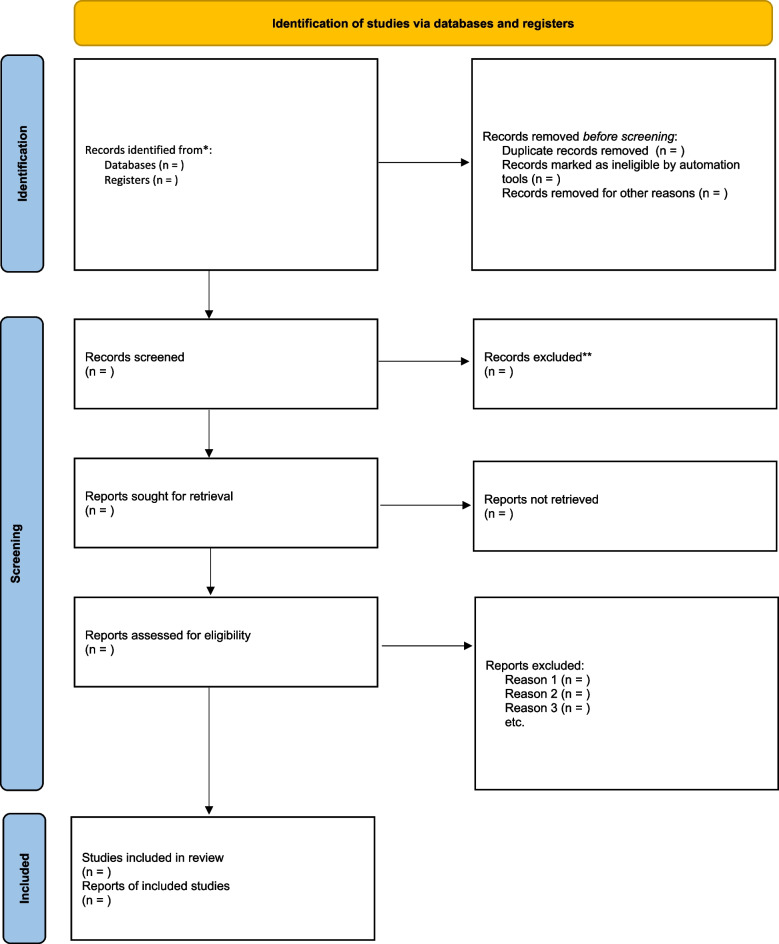
PRISMA flowchart for study selection. Identification of studies via databases and registers

The NHMRC body of evidence matrix will be used to guide the overall synthesis of findings. This allows recommendations to be formed that indicate the strength of the body of evidence in relation to the research question. Grades range from A to D, with A or B recommendations generally based on evidence that can be trusted to guide clinical practice. Grades C or D must be applied carefully based on individual clinical circumstances and should be interpreted with care [42]. The resulting data will be reported following PRISMA guidelines (Fig. 2). This figure will be used to report the number of citations at each stage of screening and selection.

## Discussion

To date, this will be the first systematic review on the use of AI in predicting the need for prostate biopsies based on clinical and demographic indicators. This systematic review will summarise the AI techniques including the ways in which they are used, their effectiveness, and their impact on clinical practice. The analysis of the literature will be used to assess evidence of AI application in clinical practice for prostate biopsy patient selection. It is expected that the findings will inform further research to address evidence gaps and to contribute to improved clinical practice.

## Data Availability

Data sharing is not applicable to this article as no datasets were generated or analysed during the current study.

## Footnotes

1 Life-threatening cancer is also known as aggressive cancer or clinically significant prostate cancer (csPCa). Prostate cancer with a Gleason score > 6 non-life-threatening cancer is also known as non-aggressive cancer, low-grade cancer, non csPCA, or indolent cancer.
